# “It’s Not Healthy to Be Too Large”—A Qualitative Study Using Participatory Methods to Explore Children’s and Adolescents’ Perspectives on Obesity Treatment and Body Image

**DOI:** 10.3390/children12101353

**Published:** 2025-10-09

**Authors:** Tove Langlo Drilen, Trine Tetlie Eik-Nes, Rønnaug Astri Ødegård, Ellen Margrete Iveland Ersfjord

**Affiliations:** 1 Department of Clinical and Molecular Medicine, Faculty of Medicine and Health Sciences, Norwegian University of Science and Technology, 7491 Trondheim, Norway; ronnaug.odegard@ntnu.no; 2 Center of Obesity Research, St. Olavs University Hospital, 7006 Trondheim, Norway; 3 Department of Neuromedicine and Movement Science, Faculty of Medicine and Health Sciences, Norwegian University of Science and Technology, 7030 Trondheim, Norway; trine.t.eik-nes@ntnu.no; 4 Nord-Trøndelag Hospital Trust, Levanger Hospital, 7601 Levanger, Norway; 5 Department of Health and Nursing Sciences, Faculty of Health and Sport Sciences, University of Agder, 4630 Kristiansand, Norway; ellen.ersfjord@uia.no

**Keywords:** pediatric obesity, children, adolescents, treatment, body image, participatory methods

## Abstract

Background/Objectives: Qualitative child-centered research on pediatric obesity treatment and body image remains limited. This study aimed to explore children’s and adolescents’ experiences with hospital-based obesity treatment and how these experiences relate to body image. Methods: A full-day workshop including three main participatory tasks was conducted in two groups of children (9–13 years) and adolescents (14–18 years), focusing on their experiences with obesity treatment and body image. Data were audiotaped, transcribed verbatim, and analyzed using reflexive thematic analysis. Results: Four main themes emerged, reflecting different aspects of participants’ experiences. The first theme, Talk with me and not my parents, encompassed participants’ desire for greater agency, as children described lacking information and feeling excluded from consultations. The second theme, Experiences of communication with healthcare professionals (HCPs) about obesity, concerned participants’ perceptions of trust, support, and non-judgmental communication, with some adolescents expressing a need for additional psychological support. The third theme, Internalization of lifestyle advice, indicated that healthy diet was viewed as the primary focus of obesity treatment, while physical activity received less attention. The final theme, Perceptions of the body, conveyed mixed experiences with weighing and most participants perceived weight loss as success in treatment and weight gain as failure. The participants shared experiences of weight-based bullying, perceived stigma, and challenges with maintaining a positive body image in a society with stereotypical thin and muscular body ideals. Conclusions: Body image was influenced by HCPs’ emphasis on health and body size, and by their own internalized perceptions, influenced by societal ideals and experiences of stigma.

## 1. Introduction

### 1.1. Pediatric Obesity Treatment

Pediatric obesity represents a global health concern, affecting more than 39 million children under 5 years of age and 390 million children and adolescents 5–18 years [1]. This places obesity among the most prevalent non-communicable disorders in childhood worldwide, with concerns related to both physical, psychological, and social consequences [2,3]. Compared to most pediatric disorders, obesity is readily identifiable by others due to increased body size, making it particularly susceptible to stigma and negative social attitudes [4,5]. Consequently, children with obesity experience and report weight-based bullying more often than their peers [6], which can contribute to body dissatisfaction and elevate the risk of disordered eating [7,8], other mental issues, and further weight gain [9].

The current guidelines and gold standard for pediatric obesity treatment comprise a family-oriented, multi-component, and multidisciplinary behavior change approach, typically including diet, physical activity, and behavioral components [10,11,12]. Pediatric obesity is recognized as a sensitive issue, and there has been a shift away from focusing primarily on weight and fat mass reduction towards approaches that are more sensitive and less stigmatizing, prioritizing overall health [13,14]. The effectiveness of obesity interventions to date has been reported as low to moderate [15], with high attrition rates observed in previous studies [16,17,18]. Variation in attrition and intervention outcomes has been attributed to factors such as insufficient psychological and emotional support [19], discrepancies between parental and clinical expectations [20], as well as parents’ efforts to protect their children from stigma and negative body image [21].

Weight-related conversations with children and their families have been reported as challenging by both parents [22] and healthcare professionals (HCPs) [23], with primary concerns regarding potential negative impacts on children’s body image [13,21]. In contrast, some quantitative studies suggest that multidisciplinary pediatric obesity treatment can improve self-esteem and body image [24,25,26], while others argue that potential harm is underexplored [27]. A qualitative review underscores the need to examine children’s and adolescents’ perspectives on obesity treatment to ensure interventions are psychologically supportive, safe, and responsive to the needs, expectations, and lived experiences of young people with obesity [28]. However, children’s perspectives remain underexplored in this context [20,29,30].

### 1.2. Children’s Voices in Pediatric Obesity Research

Following the United Nations Convention on the Rights of the Child (UNCRC), which affirms children’s right to express their views on matters affecting them, including their healthcare [31,32], the importance of integrating children’s perspectives into health research has been increasingly recognized [33,34,35]. Yet, children’s voices in pediatric obesity treatment are often mediated through parents or HCPs [20,36,37,38,39], reflecting an assumption that children lack the capacity, vocabulary, or preferences to provide meaningful input about their care [35]. Research shows that pediatric patients are both willing and capable of providing valuable feedback on their healthcare experiences [40,41,42]. Importantly, children and adolescents may perceive healthcare differently from parents and HCPs [16,35], and some are more willing to discuss sensitive issues, such as negative feelings about themselves, when asked without their parents present [39], highlighting the value of including children in research independently to capture their unique perspectives [43].

The voices of children with obesity remain underrepresented [39,44], particularly among the youngest age groups [35], and only a few studies have explored children’s unique experiences with hospital-based pediatric obesity treatment [16,29,45,46]. Existing studies show considerable variation in how children and adolescents perceive obesity treatment [46], including positive experiences related to weight loss [47], improved self-esteem, social support [48], practical guidance on healthy eating and physical activity, and appreciation of having someone to talk to about weight and health [16]. Conversely, negative experiences were related to inconsistent parenting, lack of parental support [48], and a discrepancy between expected and actual weight loss [45]. To date, there is a lack of qualitative research exploring children’s perspectives on obesity treatment and how this might relate to their body image.

In health research, children and adolescents are often included as informants through surveys, questionnaires, or semi-structured interviews with predefined topics, typically with limited participation and influence on the research, often alongside their parents [17,20]. By contrast, within childhood studies, children are viewed as embodied health actors and competent interpreters of their social world(s) [49,50], with participatory methods being emphasized as a promising way to elicit richer insights into sensitive topics [34]. Such approaches have been used with pediatric patients living with asthma, cancer, diabetes, HIV/AIDS, and disabilities [33,38,51], generating valuable knowledge about their challenges and coping strategies [52,53]. However, participatory approaches have rarely been used in pediatric obesity treatment [39,54].

### 1.3. Children’s Voices on Body Image in Obesity Care

Body image concerns are closely linked to obesity and are often associated with stigma, discrimination, and victimization [8]. Children and adolescents frequently report negative experiences and concerns related to their bodies, and obesity is widely recognized as a highly sensitive and stigmatized condition [3]. Quantitative studies show higher levels of body dissatisfaction among individuals with obesity compared to their peers in lower weight categories [7], and qualitative studies have confirmed this finding [55,56]. Despite this, few studies have examined how body image is shaped and experienced within the context of obesity treatment, particularly among children and adolescents with severe obesity.

Existing research suggests that children and adolescents value opportunities to discuss body image with HCP [16] and express a need for greater attention to psychological challenges [57]. However, studies lack meaningful child participation, particularly in hospital-based settings [39]. When participatory, child-friendly methods are used, body image often emerges as a sensitive yet important concern, typically framed in relation to social stigma, teasing, and peer acceptance rather than health. These findings highlight the need for further research to understand how children with obesity experience body image during treatment and to inform more supportive, responsive, and person-centered care.

### 1.4. Research Gaps and Study Aim

Despite growing interest in involving children and adolescents in healthcare research, few studies have examined their perspectives on pediatric obesity treatment or how body image is shaped, experienced, and influenced in this context. This study aims to address this gap by exploring how children and adolescents with severe obesity experience obesity treatment, and how these experiences relate to body image.

## 2. Method

### 2.1. Study Design

In this qualitative study, we used child-friendly, participatory methods inspired by the framework of childhood studies to explore the participants’ views on obesity treatment and body image. Consistent with childhood studies, the aim was to do research *with* rather than *on* the participants, ensuring their voices were heard [50].

### 2.2. Recruitment and Characteristics of Participants

Participants were purposively recruited from a hospital-based obesity clinic in Norway between September 2022 and January 2023. Inclusion criteria were children and adolescents aged 9–18 years who had received specialized obesity treatment for at least six months. Eligible participants were invited via mail, clinic flyers, or direct approach by HCPs. Interested children (with parents) and adolescents attended individual pre-meetings to receive detailed study information and establish a relationship with the researcher (TLD). Of 12 eligible participants, two were unavailable on the day of data collection, and one became ill the same morning, resulting in a final sample of 9 participants (two males), aged 9–18 years. The wide age range was selected to capture variation in pediatric obesity treatment experiences. All participants were White/Caucasian, except one who identified with a different European ethnic background. They represented both urban and rural areas of different socioeconomic status, with 2–10 years of experience in pediatric obesity treatment. All but one participant were still living with obesity.

### 2.3. Data Collection

Data were collected during a one-day workshop arranged at an obesity clinic in February 2023. The workshop was conducted from 10:00 a.m. to 5:00 p.m. on a Saturday, with no HCPs present. None of the researchers involved in the data collection were affiliated with the obesity clinic. The parents of the participants were absent during the data collection to ensure that the views of the children and adolescents were uninfluenced. Inspired by methods often used within childhood studies, a Mosaic approach was used [58], which combines multiple participatory methods to explore children’s perspectives about obesity treatment and body image from various angles. This approach reflects the view of children as active participants and competent interpreters of their social worlds [49].

Three main participatory tasks were developed and age-adjusted to elicit children’s views on obesity treatment and body image. Due to the wide age range, participants were divided into two groups: children (9–13 years) and adolescents (14–18 years). A step-by-step workshop guide was prepared for the researchers. All methods and the content of the workshop were reviewed and approved by four youth experts aged 14–18 years (two males and two females) with prior experience of obesity treatment. The children’s group was moderated by TLD (PhD candidate, clinical dietician), and the adolescents’ group was moderated by TTEN (PhD, specialist in eating disorders). Field notes were taken by two anthropologists (PhD), both trained in ethnographic methods, including EMIE. An additional researcher (MSc Nursing) facilitated and safeguarded the children’s group.

### 2.4. Participatory Tasks

Except for the introductory warm-up and a joint summary at the end, three main participatory tasks were conducted. Detailed information about all the participatory tasks can be found in Supplementary Materials S1.

#### 2.4.1. Photo Memory Task

In the first participatory task, photographs were used to elicit memories related to (a) the first hospital visit, (b) measurements at the clinic, and (c) communication with healthcare professionals (Table 1). Each photo session was initiated with a five-minute individual brainstorming exercise, where participants wrote their associations and experiences on Post-it notes. At the end of each photo-memory task, the Post-it notes were placed on a common worksheet and discussed within the groups. Children were also offered emojis to express their feelings.

Both groups completed the same photo-memory tasks, except for (c) communication with healthcare professionals. In this case, the children engaged in role-play, simulating a hospital consultation. The children organized the role-play and assigned roles to both adult and child participants (e.g., as patient, parent, HCP, or other).

#### 2.4.2. The Board Game Task

The second participatory task involved a Norwegian board game [59] with associated conversation cards about body image and self-esteem [60], which were adapted to fit the aim of this study. Participants rolled dice and moved to either a square depicting an emotion (sad, glad, angry, scared, like, don’t like), which they were asked to associate with experiences from obesity treatment, or to a question mark, which corresponded to 15 adapted conversation cards (e.g., “How do you think it will be for a person to hear: ‘You are too big!’). All players were invited to comment on each question, with the option to abstain, respond from either their own experiences, or share what they thought others might feel. Perspectives related to emotions in obesity treatment and to the conversation cards on body image were written on Post-it notes and placed on two separate posters, which were discussed continuously with the participants.

#### 2.4.3. Ranking Task

The purpose of the third participatory task was to compile the most important results and further facilitate discussions within the groups. All participants were asked to rank and discuss three positive and/or negative elements of obesity treatment and were further invited to suggest improvements.

### 2.5. Data Analysis and Reporting

We employed an inductive reflexive thematic analysis, following the principles of Braun and Clarke [61]. Multiple modes of data were collected, including ethnographic fieldnotes, posters, and drawings produced during the participatory tasks. All sessions in both groups were audio recorded and transcribed verbatim by TLD.

To ensure a reflective and transparent process, all four authors contributed to the analysis. First, two authors (TLD and EMIE) familiarized themselves with the data and developed a coding book containing data extracts, interpretive descriptions, and preliminary main and subthemes. TLD then systematically coded the entire dataset using this framework, organizing the material in Microsoft Word tables (Table 2), with different colors to distinguish children from adolescents’ data.

TLD and EMIE met frequently to review code labels and themes across the dataset, ensuring a recursive process of coding and theme development. Thematic maps were used to visualize main themes and subthemes, which were continuously reviewed and refined. All authors participated in the theme revisions. When coding discrepancies arose, we explored multiple interpretations of the data to refine the coding framework. These discussions were used to deepen our understanding and to make informed decisions about how best to present participants’ experiences. Final themes were defined and named through iterative team discussions and refinement of theme labels. 

The manuscript preparation adhered to the Consolidated Criteria for Reporting Qualitative Research (COREQ checklist), as seen in Supplementary Materials S2 [62].

### 2.6. Ethical Considerations

The study was evaluated by the Regional Ethics Committee in central Norway (REC Central) and approved by the Norwegian Agency for Shared Services in Education and Research (SIKT) on 19 September 2022 (no. 651514). Written assent and consent were obtained from all the participants and parents or legal guardians of children under the age of 16. The participants were informed about their right to withdraw from the study at any point. The last author (RAO) was available as a medical doctor and a safeguard during the workshop. To ensure the anonymization of the two boys, no citations presented in this article are gender specific.

## 3. Results

Four main themes, with corresponding subthemes, were generated from the thematic analysis (Table 3).

### 3.1. Talk with Me and Not My Parents

The first main theme highlighted participants’ perceived lack of agency in obesity treatment. Across the sample, participants reported receiving insufficient information before starting treatment. Particularly, children expressed frustration at feeling overlooked and dominated by their parents, emphasizing their desire for greater involvement in decisions about their own treatment.

#### 3.1.1. The Unspoken Words Before the First Visit

Most participants reported that they had insufficient information at the first hospital visit; they had not received or seen any invitation letter from the obesity clinic, and only a few recalled conversations with their parents beforehand. The majority were unaware of the reason for their hospital visit and did not appear to be involved in the decision-making process, exemplified by this quote:

*P7: “I think it was Mum who wanted me to start here [hospital] (…) I didn’t know what I was going to do, I just went along with it.”*

*(17 years)*

The lack of information regarding the first visit seemed to trigger several emotions among the participants, such as fear and uncertainty about not knowing what would happen:

*P4: “I didn’t know if it was something [wrong with me], she [mother] just said I was going to the doctor, so…”*

*(13 years)*

*P1: “[I was] a little bit nervous about what was going to happen.”*

*(9 years)*

*P5: “I was a little nervous because we were not told what to do.”*

*(14 years)*

#### 3.1.2. Feeling Excluded During Consultations

A feeling of being excluded was also reported by all child participants in conversations with HCPs and their parents. They experienced that the parents were given too much space in the consultations, giving little space for the children to participate:

*P2: “Suddenly, your parents just start talking, and you get bored…”*

*(10 years)*

*P1: “Talking about completely different things than what they are supposed to.”*

*(9 years)*

*P4: “Then you don’t understand anything about what’s going on.”*

*(13 years)*

**Figure 1:**
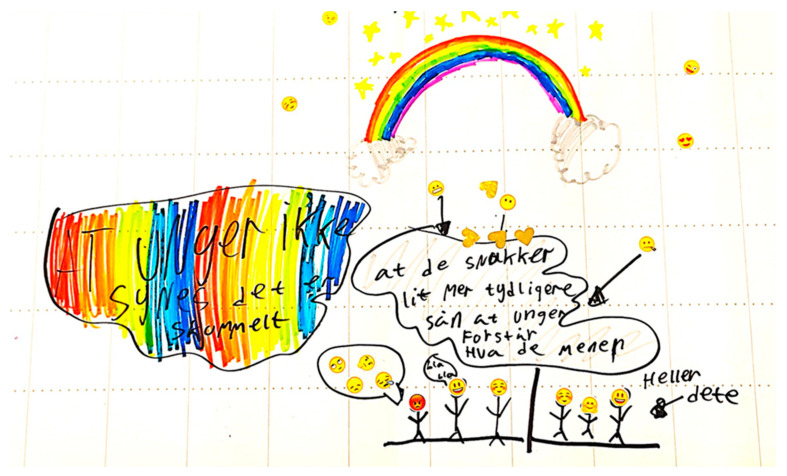
An illustration made by a child when asked to suggest improvements to obesity treatment. **Left comment:** That children don’t find it [obesity treatment] frightening. **Right comment 1:** That they [HCPs] talk more clearly so that children understand what they mean. **Right comment 2:** Rather this (and drawing of an arrow).

One child selected an emoji with a zipper mouth to visually describe the frustration of being left out of conversations. Another child illustrated parents and HCP talking to each other, with a furious emoji expression on the child’s face when being left out and a happy face when being included (Figure 1).

#### 3.1.3. Wanting to Be at the Center of Consultations

The children argued that they should be at the center of the discussions about their treatment:

*P2: “We [children] are the ones who should tell [HCPs] what we could be better at, and then they ask the parents instead when it’s supposed to be us [to be addressed].”*

*(10 years)*

*P1: “We can tell what’s in the lunch box ourselves.”*

*(9 years)*

*P3: “They [HCPs] did it [asked for permission to talk to the parents] the first time. But later, they just spoke with my parents, without asking me. So, I am just like, okay, fine, so then you do not need me to be here then!”*

*(12 years)*

During the role-play, one child participant acting as an HCP tried to shift the focus from the parents to the child in a staged consultation:

*P1: “Now you [mother] must hush! HUSH! Be quiet, I want to talk to the patient [child]”, and “You [mother] can leave the room and wait in the corridor [so that I can talk to the child].”*

*(9 years)*

### 3.2. Experiences of Communication with HCPs About Obesity

The second theme focused on participants’ experiences communicating with HCPs about obesity. They emphasized the importance of trust and valued supportive, non-judgmental interactions. Several adolescents also expressed a need for additional psychological support.

#### 3.2.1. The Importance of Trust

Besides describing the feeling of being excluded from conversations among parents and HCPs, the participants shared that they appreciated the consultations with HCPs at the obesity clinic. Most participants described relief after the first visit and perceived that the ‘doctors’ [HCPs at the clinic] were both nice and capable of helping them:

*P2: “It was not scary at all [to go to the hospital], you just had to say it [talk about obesity]. And then they could perhaps help you with the problem. They [HCPs] were nice.”*

*(10 years)*

*P7: “…I have received help with my problems [from someone who knows this], and they have listened to me in ways that my mom and others haven’t.”*

*(17 years)*

Furthermore, the adolescent participants shared that it was important to speak with the same HCP to obtain trust, which was underlined as an important prerequisite for them to share sensitive topics:

*P6: “I have been to the same ‘doctor’ [HCP] for several years, which makes it easier to trust them. I know how they are and how they react to good and bad news [about my lifestyle changes].”*

*(14 years)*

*P5: “… I feel that if I trust them [HCPs], then I know how they will react. And whether they will understand me or not.”*

*(14 years)*

*P8: “If I have a terrible period, I might start crying. Normally, my ‘doctor’ [HCP] will hug me to calm me down, then we can talk about it.”*

*(18 years)*

#### 3.2.2. Healthcare Professionals and Their Neutral Approach

The most appreciated communication with HCPs at the obesity clinic was their encouraging and ‘neutral approach’ focusing on overall health and well-being, rather than solely on weight loss:

*P8: “Here they [HCPs] are quite kind, encouraging you to achieve your goals.” *

*(18 years)*

*P9: “I felt that they [HCPs] managed to stay quite neutral… Often if I’ve gained weight, they didn’t show any big reactions or facial expressions. They often try to ask about how I feel first.”*

*(17 years)*

*P6: “Yes, and they were also trying to sort of … If there was bad news … to try to get it as positive as possible.”*

*(14 years)*

#### 3.2.3. Suggestions for Improvement

Despite all participants being satisfied with HCP’s ‘neutral approach’, they wanted to be listened to and to be a central part of communications with HCPs. The younger children underlined that HCPs should ask them for permission before talking to their parents, to ensure they were included in the conversations. As suggested by one child:

*P1: “Talking to them [children] about a few questions and then asking the mother and father one or two questions, and then back to the kids again. Then they [children] will get the opportunity to answer as much as possible, instead of the adults answering everything.”*

*(9 years)*

This was supported by the adolescent participants, who wished for more agency in decisions about parental involvement in conversations:

*P7: “If Mum comes in, she usually tells the doctor [how I am doing]. But very often she is told to go out… I don’t know why, but I feel safer when it’s just me and the doctor [HCP]. I know that no matter what happens, they have a duty of confidentiality.”*

Additionally, the adolescent participants emphasized the need for more time to talk about psychological issues and recommended that this should also be available for young children in obesity treatment. One adolescent participant (P5) shared: *“I think it’s important that you can get a psychologist or health worker here, who we can talk to.” (14 years)*

### 3.3. Internalization of Lifestyle Advice

The third theme emphasized diet as the central aspect of obesity treatment. Children focused on nutritional details, such as food choices, portion control, and adequate nutrient intake, while adolescents described a more complex and emotional relationship with food. Although physical activity was acknowledged as beneficial for health, it received less attention and was, by some adolescents, considered less important for obesity treatment than diet.

#### 3.3.1. Emphasis on a Healthy Diet

Food was a central topic of the participants’ discussions. All emphasized the importance of maintaining ‘a healthy diet’ as part of obesity treatment, and reproduced the HCP’s messages during consultations about ‘healthy eating’ that included messages about food choices, regular meals, and portion control:

*P2: “I came here [to the hospital] to talk about not eating so much food.”*

*P1: “One can try to have more vegetables in the lunch box, to eat healthier.”*

*P3: “Take the four meals that you must have; breakfast, lunch, dinner, and supper.”*

*P5: They [HCPs] wonder about what we have eaten, and learn more about our individual problems of obesity, and then we talk about the problem and how we can deal with it.”*

Moreover, ‘What they eat’ was suggested by several child participants as the second most important question that the HCPs should ask them about, following how they are, and all participants indicated that reducing the intake of sweets was one of the most important ‘dietary advice’ in obesity management. During their role-play, one child participant elaborated on how sweets may affect health, using a dog as an example:

*P1: “If you give your dog too many treats, it might be sick, and you must take it to the veterinarian. […] It [the dog] must eat sufficient nutrition to survive […] Dogs cannot live on treats only, then it will be a very unhealthy dog.”*

The children also had interesting discussions about the ‘appropriate’ amount of food, suggesting that many had knowledge about the importance of limiting their food intake and at the same time ensuring ‘sufficient’ intake:

*P2: “They [HCPs] say that you can take some food until you feel moderately full, just don’t eat too much.”*

*P4: “Take less [smaller servings] and eat slowly.”*

*P1: “That there is enough food so that I don’t have to be so hungry. For dinner, I always want more food than necessary, because I still feel hungry, because I think the food is so good, and then I want more even when I’m full. But eating too much is not healthy.”*

The adolescents emphasized more individual reflections and emotional responses when discussing dietary advice from HCPs than younger children:

*P8: “Continue to have a good relationship with food. You can still enjoy yourself, particularly on Saturdays.”*

*P9: “For instance, if you want to lose weight, you should not avoid everything unhealthy. It is about what you eat, but also how much you eat. You can eat pizza if you don’t eat too much.”*

*P7: “I was advised not to think too much about what I ate because I started overthinking.”*

#### 3.3.2. Less Focus on Physical Activity

The role of physical activity was also mentioned in the participants’ discussions; however, it was given much less attention than dietary advice. Being physically active was associated with success and linked to well-being, muscular strength, and improved mental health, but seemed to be understood as less associated with weight regulation than diet:

*P9: “I knew why [I gained weight]. It was because I was eating too much. I have always exercised a lot and stuff, but when you don’t pay attention to what you eat, it doesn’t matter how much you exercise […]”*

### 3.4. Perceptions of the Body

The fourth theme conveyed mixed experiences with weighing, and several participants perceived weight loss as success in treatment and weight gain as failure. All participants shared experiences of weight-based bullying, and the majority perceived stigma and challenges with maintaining a positive body image in a society with stereotypical thin and muscular body ideals.

#### 3.4.1. The Ambivalence of Body Size Assessment in Obesity Treatment

Height and weight measurements performed to assess and monitor body mass index (BMI) and growth development seemed to be understood as a central part of the obesity treatment, and were commonly discussed among the participants:

*P1: “This [height and weight measure] is the first thing I do every time I come here.”*

*P3: “They [HCPs] used to show me a bow [BMI growth chart]. Sometimes it went down, and last time it was straight. They say: “If we continue to do things the same way, it [the weight status] can be better.”*

Nearly all participants expressed enthusiasm for the height assessment, and growing taller was viewed positively. One child participant said: *“I thought it was fun and exciting to know my height.” (P2)* and was supported by an adolescent participant: *“I was always looking forward to it [the height measure] … because I was always growing and enjoyed being taller” (P6)*. Only one adolescent expressed negative experiences with the height measurement due to being teased for short stature:

*P7: “I did that [dreaded being measured] because I shrank and grew downwards instead of upwards. I used to be the tallest in third grade, and then suddenly I was nicknamed for my [low] height.”*

By contrast, weight assessment was discussed with less enthusiasm than the height measure and elicited mixed feelings. The youngest participants, however, seemed confident about being weighed and used a smiling emoji when describing the weighing experience. One child said: *“I thought it was fun to stand on that weight because it talked instead of just showing it [the number] on the screen” (P1)*. Most of the older children and adolescent participants indicated ambivalence and more negative perceptions of being weighed: *“You can see it [the weight] on the screen.” “You remember it.” You compare it with your previous weight” (P6)*. One child participant drew a sad person on a scale when asked to describe the least enjoyable experience from obesity treatment, reflecting the disappointment when the number on the scale continued to increase, despite efforts to reduce weight. During roleplay, the same child covered the numbers with his feet during the weighing procedure.

Further discomfort was described by one adolescent girl who developed a strained relationship with her body weight after too frequent weighing: *“At one point I did it [weighed myself] so often that I wasn’t allowed to do it anymore [by the HCPs]. I had to check [my body weight] all the time” (P7)*. Some adolescents also shared that they weighed themselves between the consultations, and some were fasting on the same day. Despite several negative experiences related to weighing, a few shared ambivalence or positive experiences:

*P5: “I like it a little bit that they tell the weight to parents and us. Because then we’ll hear how we’re doing, so that’s positive. But it’s not always fun […] “You are standing there, waiting to know your body weight.”*

One adolescent claimed to develop a more relaxed relationship with weighing as time passed by:

*P9: “I do it [weigh myself] every day when I get up [from bed], it has become a habit. Previously, I did it because I knew [that the weight had gone up]. Now, there is no stress […] I have a healthier relationship with my weight and monitor it weekly.”*

#### 3.4.2. The Paradox of Perceived Success in Treatment—Healthy Body or Weight Loss?

Initially, perceived success in obesity treatment was described with positive emotions linked to healthy lifestyle changes, whereas perceived failure was associated with negative feelings and difficulties with adhering to the recommended advice:

*P7: “A good period is when you focus on eating the correct food and manage to be physically active. A bad period can be when you feel bad about yourself and feel like doing nothing, eating the first thing you see…”*

Further into the discussions, the perceptions of success or failure in obesity management seemed to be more closely linked with body weight status, with positive feelings being evoked when the weight was reduced, and more positive feelings were described when their weight was increased:

*P9: “It also became a moment after a while, so that you became a little extra stressed if you knew that you had gained weight.”*

*P4: “That you don’t get very good comments on what you’ve tried to do. That can make you very sad. Or that you try your best but don’t see any results [weight gain].”*

By contrast, most participants agreed that being healthy was more important than losing weight, and some claimed that not all weight gain was unhealthy: *“So even if the curve on that screen has increased, that does not necessarily mean a bad thing, that I haven’t done my effort. It might as well be that I have grown…” (P5)*. However, the discussions revealed a dilemma in which weight loss was closely associated with being healthy. This was illustrated by one adolescent participant criticizing the acceptance of larger body sizes in social media:

*P9: I don’t think it’s good to standardize and idealize people who are far too large, and the media makes it okay. […] You should be healthy, and it’s not healthy to be too large; that’s just unhealthy.”*

#### 3.4.3. Perceptions of Stigma

The issue of stigma was a recurring theme in participants’ conversations, including body size-related bullying, the shame of going to obesity treatment, and a sense of being judged in the context of follow-up conversations. For example, one child participant used an emoji of a face with wide-open eyes and no mouth to indicate early experiences with talking about obesity with a HCP: *“I was just silent. And you think a lot about what they [HCPs] say. Then you’re very quiet, while you’re listening, and you’re a bit, like you don’t want to talk about it so much.” (P2)*

All the participants described situations of being bullied, and most adolescents said that they could still remember the negative and painful experiences: *“You get hurt on the inside, and everything falls apart” (P6)*. The children described in detail how they had been bullied because of their body size, or being told that they were too fat:

*P2: “Someone said that [You are too fat] to me in first grade.”*

*P3: “It happened to me as well, from grade 1 to 5.”*

*P4: “It happened so often to me that I stopped caring about it.”*

A range of emojis (tears, squeezing their eyes shut, with a headband-aid, or snorting out of the nose) were used by the child participants to indicate feelings of sadness, anger, and discomfort in response to being bullied. Simultaneously, several strategies to cope with bullying were shared, such as hiding their feelings or being physical:

*P3: “I do not show any reactions.”*

*P4: “I tried that once [not showing any reactions], but he [the bully] just kept on going, even after I said stop. He started to bother me even more, but then I hit him in his stomach, and he stopped.”*

Several adolescent participants described shame about attending an obesity treatment clinic. They did not use the name of the clinic, and most of them lied or made excuses about where they were going:

*P9: “I remember that I’ve always tried to hide it. If someone asked: “Oh, where have you been?” Then I always said: To the doctor. I didn’t say the Obesity Clinic or anything like that. Maybe because I felt ashamed about going here.”*

*P7: “I told my friends, but didn’t dare to tell anyone else.”*

*P5: “I say that I am going to check my asthma.”*

The adolescent participants also associated the clinics’ waiting room with negative emotions, inducing physiological stress responses: *“Long waiting time, with more time to get stressed” […] “High pulse” (P6)*. *“Heart palpitations and heavy breathing” […] “Sweaty hands” (P8)*. One adolescent participant expressed that the degree of stress was related to expectations of success or failure:

*P9: “If I know that I will get good news, I am rarely stressed. I was often more stressed if I knew I had not done what I was supposed to, and I gained weight. I didn’t think much about it at home, but here [at the waiting room] it was more like ‘judgment day’.”*

By contrast, all the child participants shared positive associations with the waiting room, focusing on the ‘silence’, ‘nice people’, and the colorful fixtures and decorations on the walls, indicating no sense of judgment.

#### 3.4.4. Body Positivity and the Ideal Body—A Balancing Act

At the beginning of the workshop, all participants emphasized the importance of having a positive attitude towards their bodies, and the children particularly highlighted the importance of body satisfaction:

*P2: “You must be satisfied with your body.”*

*P3: “You have to believe that you are looking good.”*

*P4: “I want to be myself.”*

*P1: “Your body is perfect just the way it is, and no one else can change your body (…) without you wanting to.”*

Also, the adolescents underlined the importance of body positivity and acceptance early in the workshop:

*P6: “Think positively, be satisfied. Believe that it [body appearance] is not that important.”*

*P7: “Accept that everyone looks different.”*

Further into the workshop, the discussions amongst most participants revealed that their positive body talk was challenged by Western body appearance ideals. The female ideal body was solely perceived as thin: *“They [girls] want to show that they are thin, and nice” (P1)*. Also, the adolescent participants added more characteristics to the ideal female body: *“Big tits, a time-glass figure, and a large butt” […] “Slightly tall” (P5)*. *“Tanned” […] “Blondine or brunette” (P6)*. The ideal body size for boys was described as both thin and muscular: *“A lot of them want to be thin”. “They want to show the girls how strong they are […] Taking their shirt off”. “Maybe like a bodybuilder” (P9).* High stature was also mentioned as an important characteristic of the ideal male body: *“I will say height, for sure. People want to be tall. There’s a lot in the media about females saying that they will not date a boy below 1.80 cm/6 feet” (P9)*.

Both child and adolescent participants verbalized the difficult balance between body positivity and societal ideal bodies, indicating a personal desire for a thinner and more muscular body size:

*P3: “I want to change my body. I want to become the old me… when I used to be thin.”*

*P4 “[I like it when] … things are going in the right direction, and I am losing weight.”*

*P9: “I’m not going to lie that it would have been nice to have a six-pack.”*

## 4. Discussion

In this study, we explored children’s and adolescents’ (ages 9–18 years) experiences with hospital-based obesity treatment and how these experiences related to body image. Inspired by childhood studies [50,58], we used child-friendly participatory methods to explore their views. Our findings suggested an interplay between participants’ experiences of obesity treatment and their perceived body image: while treatment seemed to shape how children and adolescents perceived their bodies, the children’s body image simultaneously influenced how they experienced and responded to the treatment.

First, our results indicated that insufficient information before starting obesity treatment contributed to unnecessary anxiety and concerns among the participants. This lack of information has previously been reported among children in obesity treatment [36,42] and may, in part, reflect parents’ discomfort in addressing obesity and discussing the need for treatment with their children [22]. Our results suggest that the parents’ well-intended efforts to protect their children, by excluding them from conversations or decisions about their obesity treatment, may have signaled that something was ‘wrong’ with participants’ bodies. This lack of inclusion and autonomy may have contributed to unnecessary anxiety and concern among the participants.

Secondly, ‘being healthy’ seemed to be a recurring treatment goal emphasized by HCPs and was described early in the workshop by participants as the most important outcome of obesity treatment. Success was often associated with adopting a healthy lifestyle, such as eating healthy food and being physically active. Participants also underlined the value of body positivity, stressing that one should be satisfied with one’s body regardless of shape and size. However, as the workshop progressed, it became clear that most participants also desired a thinner and smaller body aligned with societal ideals. Our findings suggest that HCPs’ routine measurements of height and weight—used to illustrate progress on growth charts indicating whether participants were ‘outside’ or ‘within’ the normal range—may have reinforced societal pressures related to body size. Several participants further linked success and failure directly to body size, with weight loss associated with positive feelings of achievement and weight gain more often experienced as personal failure and negative emotions.

Our findings suggest that participants’ divergent perceptions of ‘success’ in treatment reflect their lived realities. Within the ‘hospital world,’ they encountered a potential mismatch in HCPs’ communication about the importance of ‘good health’ and the simultaneous emphasis on progress measured through growth and BMI charts. In the ‘social world’ outside the hospital, participants described repeated exposure to bullying and stigma related to their larger bodies, which negatively affected their body image.

Participants’ internalized weight stigma and body shame also became apparent when many of the adolescents shared that they lied to their peers about going to an obesity clinic because they felt ashamed. The participants, except for the youngest ones, also shared that they felt judged by others in the waiting room at the obesity clinic, further suggesting internalized shame. Weight-based bullying and social stigma of attending obesity treatment described by our participants reflect findings from other studies [30], where experiences related to bullying and stigma are portrayed as challenging, especially for children and young people living with severe obesity [46]. The younger children appeared less affected by body stigma and seemed to be more accepting of messages from HCPs and parents about being healthy and satisfied with their bodies, possibly because of their inability to reflect deeply or worry about these issues [63].

Our results indicate that the development of body image in children and adolescents attending obesity treatment is not only shaped by their treatment, but also by exposure to stigma, bullying, and the societal ideals that influence how they perceive, experience, and view their bodies. While many participants expressed a desire and an intention to feel satisfied with their bodies regardless of their size, these aspirations were often challenged by pervasive societal norms that equate thinness and muscularity with health and self-worth. As a result, the perceived success of their treatment was often equated with weight loss, even if “a healthy body” was initially identified by HCPs as the primary goal.

Despite a clear shift in clinical guidelines toward a health-centered approach in pediatric obesity treatment [13,14], our findings indicate that the philosophy, goals, and methods applied in practice are still perceived as weight-centered by most participants. This implementation gap may be unintentional, reflecting prevailing clinical norms and attitudes toward the importance of weight in obesity treatment. Our study highlights this gap by incorporating perspectives from children and adolescents who are often overlooked in treatment design and delivery of obesity treatment. These insights are crucial for aligning practice more closely with guideline recommendations, fostering a more health-centered and less weight-centered approach to obesity treatment.

## 5. Strengths and Limitations

The first main strength of this study is the use of participatory methods to provide in-depth perspectives from children and adolescents about the sensitive topics of obesity and body image. Using different methods to explore this topic from different angles during a whole day in a safe environment produced rich data and gained deep insight into the participants’ perspectives. The second strength was to involve four youth experts in the development of the participatory methods used. A third strength was the multidisciplinary expertise of the research team, encompassing medicine, eating disorders, clinical nutrition, and social sciences. To enhance credibility and trustworthiness, all researchers contributed to the analysis, and interpretations were continuously discussed and refined within the team before reaching consensus. Finally, a strength is that the sample included nine participants, encompassing a diverse group of hard-to-reach children and adolescents (aged 9–18 years) across sex and socioeconomic backgrounds, providing varied perspectives and reflecting the gap in age amongst patients receiving treatment at the clinic. Although the findings may not capture the full range of children’s and adolescents’ perspectives, the experienced interviewers fostered open dialogue and generated valuable insights within a field with limited research.

A limitation in this study is the small number of boys, which may undermine their perspectives and preclude gender comparisons. Also, this study reflects the experiences of a limited group of children and adolescents in hospital-based pediatric obesity treatment. The findings provide in-depth insights that may be transferable to similar contexts, though not necessarily to all treatment settings or populations.

## 6. Future Implications and Clinical Recommendations

Findings from this research can inform current pediatric obesity treatment in many ways. First, children and adolescents should receive age-appropriate information about obesity treatment interventions from the hospital. Parents should also inform and include them in the decision-making process before initiating obesity treatment. HCPs should ask children and adolescents about their desired degree of involvement and ensure that they are at the center of consultations, being involved in their lifestyle changes. Children, and especially adolescents, should have the opportunity to speak with HCPs privately and decide for themselves whether their parents should be involved. HCPs should address issues of body weight, size, and stigma sensitively and neutrally, while offering support to help strengthen the child’s body image. Parents should also receive training in positive body talk to better support their child.

Future policy efforts should focus on enhancing self-perception among children and adolescents and reducing weight-related stigma through coordinated efforts involving healthcare, schools, and public health initiatives. Such efforts should challenge stereotypes, promote body diversity, and create inclusive environments where young people feel respected and supported.

To our knowledge, this study is the first qualitative study using child-friendly participatory methods to explore how pediatric obesity treatment might influence children’s and adolescents’ body image. The findings highlight the need for further research examining how pediatric obesity treatment might affect children’s and adolescents’ body image. Employing participatory approaches is recommended to gain deeper insight, with meaningful engagement of children. Future research could also adopt a mixed-methods design, which will enable integration of quantitative outcomes with qualitative insights, providing a comprehensive understanding of children’s experiences of obesity treatment and its impact on body image.

## Figures and Tables

**Table 1:** Images used in the Photo Memory Task.

The First Hospital Visit	Measurements	Communication with Healthcare Professionals
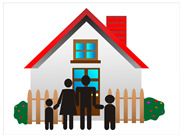 At home ^1^	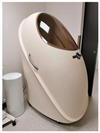 BodPod ^2^	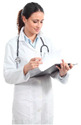 Healthcare professional
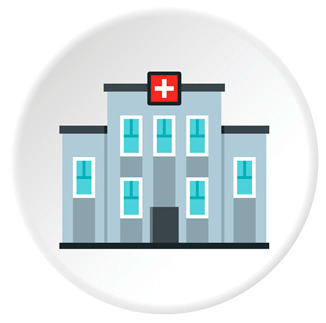 At the hospital ^1^	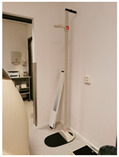 Stadiometer	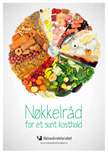 Dietary recommendations
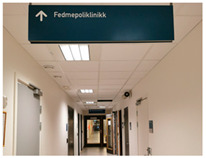 The obesity clinic	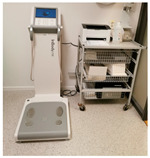 InBody	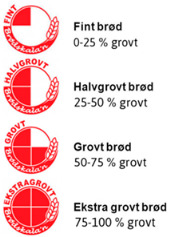 Bread scale ^3^
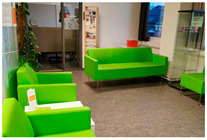 The waiting room	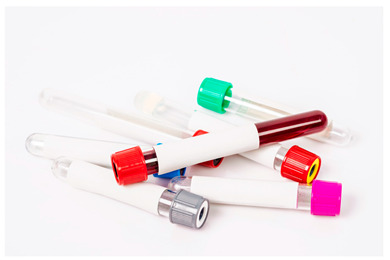 Blood tests ^1^	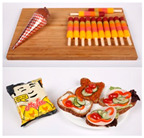 Food illustrations
	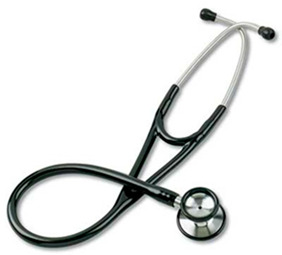 Stethoscope ^1^	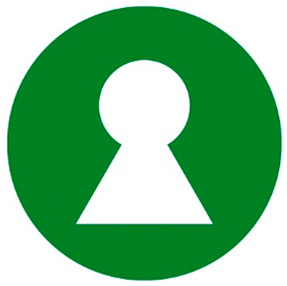 The “keyhole” symbol ^4^

^1^ Illustrations from https://www.colourbox.com (accessed on 13 September 2025) ^2^ Body composition measure based on air displacement plethysmography; ^3^ A visual scale of whole grain flour content of bread; ^4^ A symbol of healthy food choices.

**Table 2:** Examples from the coding process.

Meaning Units		Code Label	Subtheme	Main Theme
“I didn’t know if it was something [wrong with me], she [mother] just said I was going to the doctor, so…”		Lack of information	The unspoken words before the first visit	Talk with me and not my parents
“I felt that they [HCPs] managed to stay quite neutral… Often, if I’ve gained weight, they don’t show any big reactions or facial expressions. They often try to ask about how I feel first.”		Weight neutral	HCPs and their neutral approach	Experiences of communication with HCPs about obesity
“For instance, if you want to lose weight, you should not avoid everything unhealthy. It is about what you eat, but also how much you eat. You can eat pizza if you don’t eat too much.”		Food and nutrition	Emphasis on a healthy diet	Internalization of lifestyle advice
“One thin girl bullies another girl due to her larger body size because they just think that thin girls are nice and such.”		Weight-based bullying	Perceptions of stigma	Perceptions of the body

**Table 3:** Identified themes from thematic analysis.

Main Themes		Talk with Me and Not My Parents		Experiences of Communication with HCPs About Obesity		Internalization of Lifestyle Advice		Perceptions of the Body
Subthemes		The unspoken words before the first visit		The importance of trust		Emphasis on a healthy diet		The ambivalence of body size assessment in obesity treatment
		Feeling excluded during consultations		HCPs and their neutral approach		Less focus on physical activity		The paradox of perceived success in treatment—healthy body or weight loss?
		Wanting to be at the center of consultations		Suggestions for improvement				Perceptions of stigma
								Body positivity and the ideal body—a balancing act

## Data Availability

The datasets generated by the participants are not publicly available but are available from the corresponding author at reasonable request.

## Supplementary Materials

The following supporting information can be downloaded at https://www.mdpi.com/article/10.3390/children12101353/s1.

## Abbreviations

The following abbreviations are used in this manuscript:

BMI	Body Mass Index
COREQ	Consolidated criteria for reporting qualitative research (COREQ)
HCP	Healthcare professional

## References

[1] World Health Organization RofE (2022). WHO European Regional Obesity Report 2022.

[2] Jebeile H., Kelly A.S., O’Malley G., Baur L.A. (2022). Obesity in children and adolescents: Epidemiology, causes, assessment, and management. Lancet Diabetes Endocrinol..

[3] Blundell E., De Stavola B.L., Kellock M.D., Kelly Y., Lewis G., McMunn A., Nicholls D., Patalay P., Solmi F. (2024). Longitudinal pathways between childhood BMI, body dissatisfaction, and adolescent depression: An observational study using the UK Millennium Cohort Study. Lancet Psychiatry.

[4] Roberts K.J., Polfuss M.L., Marston E.C., Davis R.L. (2021). Experiences of weight stigma in adolescents with severe obesity and their families. J. Adv. Nurs..

[5] Nutter S., Eggerichs L.A., Nagpal T.S., Ramos Salas X., Chin Chea C., Saiful S., Ralston J., Barata-Cavalcanti O., Batz C., Baur L.A. (2024). Changing the global obesity narrative to recognize and reduce weight stigma: A position statement from the World Obesity Federation. Obes. Rev..

[6] Cheng S., Kaminga A.C., Liu Q., Wu F., Wang Z., Wang X., Liu X. (2022). Association between weight status and bullying experiences among children and adolescents in schools: An updated meta-analysis. Child. Abus. Negl..

[7] Moradi M., Mozaffari H., Askari M., Azadbakht L. (2022). Association between overweight/obesity with depression, anxiety, low self-esteem, and body dissatisfaction in children and adolescents: A systematic review and meta-analysis of observational studies. Crit. Rev. Food Sci. Nutr..

[8] Guardabassi V., Tomasetto C. (2022). Weight-based teasing, body dissatisfaction, and eating restraint: Multilevel investigation among primary schoolchildren. Health Psychol..

[9] Robinson E., Haynes A., Sutin A., Daly M. (2020). Self-perception of overweight and obesity: A review of mental and physical health outcomes. Obes. Sci. Pract..

[10] Tully L., Arthurs N., Wyse C., Browne S., Case L., McCrea L., O’Connell J.M., O’Gorman C.S., Smith S.M., Walsh A. (2022). Guidelines for treating child and adolescent obesity: A systematic review. Front. Nutr..

[11] Helsedirektoratet (2010). Nasjonale Faglige Retningslinjer for Primærhelsetjenesten. Forebygging og Behandling av Overvektog Fedme hos barn og Unge.

[12] Ells L.J., Rees K., Brown T., Mead E., Al-Khudairy L., Azevedo L., McGeechan G.J., Baur L., Loveman E., Clements H. (2018). Interventions for treating children and adolescents with overweight and obesity: An overview of Cochrane reviews. Int. J. Obes..

[13] Koivumäki T., Kääriäinen M., Tuomikoski A.-M., Kaunonen M. (2025). Parent and carer experiences of health care professionals’ communications about a child’s higher weight: A qualitative systematic review. JBI Evid. Synth..

[14] Jebeile H., Cardel M.I., Kyle T.K., Jastreboff A.M. (2021). Addressing psychosocial health in the treatment and care of adolescents with obesity. Obesity.

[15] Henderson M., Moore S.A., Harnois-Leblanc S., Johnston B.C., Fitzpatrick-Lewis D., Usman A.M., Sherifali D., Merdad R., Rigsby A.M., Esmaeilinezhad Z. (2025). Effectiveness of behavioural and psychological interventions for managing obesity in children and adolescents: A systematic review and meta-analysis framed using minimal important difference estimates based on GRADE guidance to inform a clinical practice guideline. Pediatr. Obes..

[16] Skelton J.A., Martin S., Irby M.B. (2016). Satisfaction and attrition in paediatric weight management. Clin. Obes..

[17] Banks J., Cramer H., Sharp D.J., Shield J.P., Turner K.M. (2014). Identifying families’ reasons for engaging or not engaging with childhood obesity services: A qualitative study. J. Child. Health Care.

[18] Barlow S.E., Ohlemeyer C.L. (2006). Parent reasons for nonreturn to a pediatric weight management program. Clin. Pediatr..

[19] Newson L., Sides N., Rashidi A. (2024). The psychosocial beliefs, experiences and expectations of children living with obesity. Health Expect..

[20] Roberts K.J., Binns H.J., Vincent C., Koenig M.D. (2021). A Scoping Review: Family and Child Perspectives of Clinic-Based Obesity Treatment. J. Pediatr. Nurs..

[21] Sjunnestrand M., Neuman N., Ek A., Nordin K., Ramos Salas X., Järvholm K., Eli K., Nowicka P. (2025). Not for children’s ears? Parents’ insights into early childhood overweight and obesity treatment. Scand. J. Prim. Health Care.

[22] Sjunnestrand M., Neuman N., Järvholm K., Ek A., Nordin K., Salas X.R., Eli K., Nowicka P. (2024). “A balancing act”: Parents’ longitudinal perspectives of weight-related discussions with their children following obesity treatment. BMC Public. Health.

[23] Abdin S., Heath G., Welch R.K. (2021). Health professionals’ views and experiences of discussing weight with children and their families: A systematic review of qualitative research. Child Care Health Dev..

[24] Gow M.L., Tee M.S.Y., Garnett S.P., Baur L.A., Aldwell K., Thomas S., Lister N.B., Paxton S.J., Jebeile H. (2020). Pediatric obesity treatment, self-esteem, and body image: A systematic review with meta-analysis. Pediatr. Obes..

[25] Eichen D.M., Strong D.R., Rhee K.E., Rock C.L., Crow S.J., Epstein L.H., Wilfley D.E., Boutelle K.N. (2019). Change in eating disorder symptoms following pediatric obesity treatment. Int. J. Eat. Disord..

[26] Epstein L.H., Paluch R.A., Saelens B.E., Ernst M.M., Wilfley D.E. (2001). Changes in eating disorder symptoms with pediatric obesity treatment. J. Pediatr..

[27] Alcoat C., Køster-Rasmussen R. (2025). Monitoring and reporting of adverse effects in weight loss trials in children. Dan. Med. J..

[28] Lachal J., Orri M., Speranza M., Falissard B., Lefevre H., QUALIGRAMH, Moro M.-R., Revah-Levy A. (2013). Qualitative studies among obese children and adolescents: A systematic review of the literature. Obes. Rev..

[29] Leaviss J., Verstraeten R., Carroll C., Booth A., Essat M., Cuevas D.C. (2025). Stakeholder views of behavioral interventions for children and adolescents with obesity: Mega-ethnography of qualitative syntheses. Obes. Rev..

[30] Carroll C., Sworn K., Booth A., Pardo-Hernandez H. (2022). Stakeholder views of services for children and adolescents with obesity: Mega-ethnography of qualitative syntheses. Obesity.

[31] UN The United Nations (1989). Convention on the Rights of the Child.

[32] Weil L.G., Lemer C., Webb E., Hargreaves D.S. (2015). The voices of children and young people in health: Where are we now?. Arch. Dis. Child..

[33] Clavering E.K., McLaughlin J. (2010). Children’s participation in health research: From objects to agents?. Child Care Health Dev..

[34] Davies C., Fraser J., Waters D. (2023). Establishing a framework for listening to children in healthcare. J. Child. Health Care.

[35] Ali H., Fatemi Y., Cole A., Tahat S., Ali D. (2022). Listening to the voice of the hospitalized child: Comparing children’s experiences to their parents. Children.

[36] Skelton J.A., Irby M.B., Geiger A.M. (2014). A Systematic Review of Satisfaction and Pediatric Obesity Treatment: New Avenues for Addressing Attrition. J. Heal. Qual..

[37] Haijes H.A., van Thiel G.J.M.W. (2016). Participatory methods in pediatric participatory research: A systematic review. Pediatr. Res..

[38] Freire K., Pope R., Jeffrey K., Andrews K., Nott M., Bowman T. (2022). Engaging with Children and Adolescents: A Systematic Review of Participatory Methods and Approaches in Research Informing the Development of Health Resources and Interventions. Adolesc. Res. Rev..

[39] Concincion S., Dedding C., Verhoeff A., van Houtum L. (2024). Building space for children’s voices: The added value of participatory and creative approaches for child-centred integrated obesity care. J. Pediatr. Nurs..

[40] Birbeck D., Drummond M. (2005). Interviewing, and listening to the voices of, very young children on body image and perceptions of self. Early Child. Dev. Care.

[41] Sevón E., Mustola M., Siippainen A., Vlasov J. (2025). Participatory research methods with young children: A systematic literature review. Educ. Rev..

[42] Schalkers I., Dedding C.W.M., Bunders J.F.G. (2015). ‘[I would like] a place to be alone, other than the toilet’—Children’s perspectives on paediatric hospital care in the Netherlands. Health Expect..

[43] Hunleth J.M., Spray J.S., Meehan C., Lang C.W., Njelesani J. (2022). What is the state of children’s participation in qualitative research on health interventions?: A scoping study. BMC Pediatr..

[44] Wyatt K.A., Bell J., Cooper J., Constable L., Siero W., Pozo Jeria C., Darling S., Smith R., Hughes E.K. (2024). Involvement of children and young people in the conduct of health research: A rapid umbrella review. Health Expect..

[45] Murtagh J., Dixey R., Rudolf M. (2006). A qualitative investigation into the levers and barriers to weight loss in children: Opinions of obese children. Arch. Dis. Child..

[46] Morinder G., Biguet G., Mattsson E., Marcus C., Larsson U.E. (2011). Adolescents’ perceptions of obesity treatment—An interview study. Disabil. Rehabil..

[47] Holt N.L., Bewick B.M., Gately P.J. (2005). Children’s perceptions of attending a residential weight-loss camp in the UK. Child Care Health Dev..

[48] Schalkwijk A., Bot S., De Vries L., Westerman M., Nijpels G., Elders P. (2015). Perspectives of obese children and their parents on lifestyle behavior change: A qualitative study. Int. J. Behav. Nutr. Phys. Act..

[49] Clark A., Moss P., Kjørholt A.T. (2005). Beyond Listening: Children’s Perspectives on Early Childhood Services.

[50] James A. (2001). Ethnography in the study of children. Handb. Ethnogr..

[51] Bailey S., Boddy K., Briscoe S., Morris C. (2015). Involving disabled children and young people as partners in research: A systematic review. Child Care Health Dev..

[52] Andersen C.S., Askheim O.P., Dolva A.-S. (2023). “We surely are researchers now!” Participatory methods as an empowering process with disabled children in research. Childhood.

[53] Hansen J.E.D., Ersfjord E.M.I. (2021). The pen, the receiver and the pump: Exploring young children’s experiences of having a parent with type 1 diabetes. Child. Soc..

[54] Larsson I., Staland-Nyman C., Svedberg P., Nygren J.M., Carlsson I.-M. (2018). Children and young people’s participation in developing interventions in health and well-being: A scoping review. BMC Health Serv. Res..

[55] Mériaux B.G., Berg M., Hellström A.-L. (2010). Everyday experiences of life, body and well-being in children with overweight. Scand. J. Caring Sci..

[56] Rees R., Oliver K., Woodman J., Thomas J. (2011). The views of young children in the UK about obesity, body size, shape and weight: A systematic review. BMC Public. Health.

[57] Rees R.W., Caird J., Dickson K., Vigurs C., Thomas J. (2014). ‘It’s on your conscience all the time’: A systematic review of qualitative studies examining views on obesity among young people aged 12–18 years in the UK. BMJ Open.

[58] Clark A. (2017). Listening to Young Children: A Guide to Understanding and Using the Mosaic Approach.

[59] (2021). Hei. Samtalekort om Kropp og Selvfølelse.

[60] Fiskum C., Eik-Nes T.T. (2023). Erfaringer med Samtalekortene «Hei Kropp og Selvfølelse»: En Kvalitativ Undersøkelse.

[61] Braun V., Clarke V. (2021). Thematic Analysis: A Practical Guide.

[62] Tong A., Sainsbury P., Craig J. (2007). Consolidated criteria for reporting qualitative research (COREQ): A 32-item checklist for interviews and focus groups. Int. J. Qual. Health Care.

[63] Harter S. (1999). The Construction of the Self: A Developmental Perspective.

