# Supplementary material for: “It’s Not Healthy to Be Too Large”—A Qualitative Study Using Participatory Methods to Explore Children’s and Adolescents’ Perspectives on Obesity Treatment and Body Image
Source: Children (Basel). 2025 Oct 9;12(10):1353. doi: 10.3390/children12101353 (PMC12562709; doi:10.3390/children12101353)
Supplement: Supplementary file 1 [file children-12-01353-s001.zip › Supplementary Materials S1_Descriptions of The Participatory Methods.pdf]

Methodology for implementing children and adolescents' perspectives on their own health service at an obesity outpatient clinic

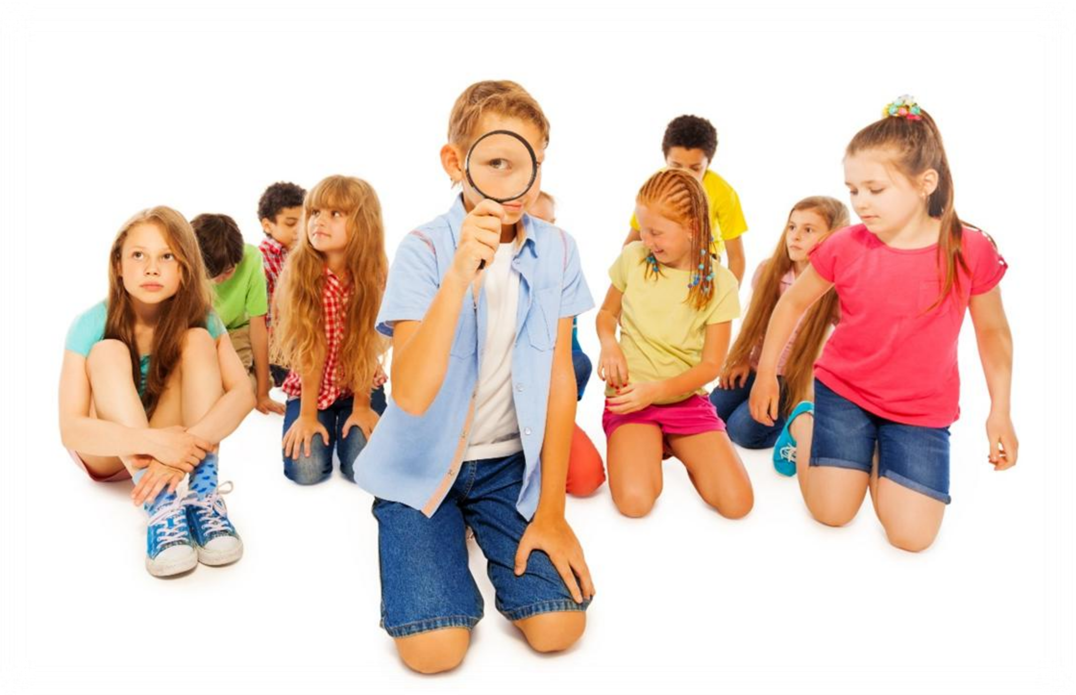

These exercises will be used to conduct a workshop with children aged 9-18 years to gain increased insight into children's perspectives on body image and their own health service for the treatment of obesity.

BACKGROUND AND IMPLEMENTATION

The data collection methods have been developed in connection with a research project where the goal is to gain a deeper insight into children and adolescents' experience of body image and their own health service for the treatment of obesity. The research question aims to shed light on children and young people aged 9-18 years' thoughts, feelings, experiences, experiences and perspectives related to their participation in obesity treatment at a hospital. The data collection methods will be child-friendly and activity-based research methods, which will be carried out in small groups together with children of the same age. The different methods will build on each other and complement each other, so that the problem is illuminated from different perspectives. The children will have the opportunity to express themselves in various creative ways.

Issues:

1. What perspectives and experiences do children and adolescents with obesity have related to participation in their own health services?

2. What are children's perspectives on body image in connection with their own obesity treatment?

Implementation:

There will be three researchers who are responsible for conducting the workshop, in addition to one person per group who takes field notes. Three exercises will be presented to be carried out in groups, where both time use and approach of the exercise will be adapted to the children's age. Notes will be taken during the workshop, and the conversations will be recorded on an audio recorder. The audio files will be transcribed into text and then analyzed by thematic analysis. The results will be published in a current scientific journal.

P.S. The methods we have presented here will be further developed and adapted to different age groups.

Warm-up exercises

The purpose of these exercises is to make the informants better acquainted, to minimise the power relationship between children and adults, and to make the setting for the research harmless.

Time: Approx. 10 minutes for each exercise, a total of approx. 30 minutes.

Preparation: Have one ball available, or three if three groups. Everyone should be involved, including the adults.

1. NAME GAME

Everyone stands in a circle. A person starts with a ball, says their name, and throws the ball to the person standing next to them. First, a full round. Then another round where the first person says their name, while the next person has to remember the previous one before saying their own name, before the ball is thrown on. The next person starts by saying the name of person no. 1, then 2 and then their own. It continues like this until the entire round is completed. If there are small children, then the groups should be smaller.

Eventually, you can add an adjective, a fruit or an animal that starts with the same initial as your name.

2. SET-UP EXERCISE

One person is selected to lead the exercise. The children are asked to line up in order of age, height and alphabetically by name. Children and young people can make their own suggestions here. Here you can get an extra challenge in that it is not allowed to talk, only allowed to use gestures and body language. Suggestion: First height without talking, then age and shoe size.

On October 3, 201 NAME BINGO

Make a bingo board consisting of 3x3 or 4x4 squares. Each box should contain something about a person, either a personal trait/something you have done or like to do. For younger children, it can be pictures. For example: Red hair, Brown eyes, T-shirt, White socks, Jewelry, Headwear, Glasses, Lenses, Have a dog, play handball, are an only child, etc. Each person gets a bingo card each, and has to go around the room and find a name that fits a characteristic. For example, if Trude has red hair, then the name Trine can be written in the box where it says red hair. Each name can only be used once.

The first one to get 3 or 4 in a row gets bingo first. Or you can keep going until the entire grid is filled in.

Suggestions for extra exercises

GETTING-TO-KNOW-YOU GAME

All the children stand in a circle or possibly be divided into smaller groups. An adult starts by asking a question and throws the ball to a person of their choice. The person who catches the ball answers the question, asks another question and throws the ball on. Some examples of questions: Do you have any pets? What's your favorite color? How many of you are in the family? What do you like to do best when it's summer?

STORYTELLING

Everyone sits in a circle or around a table. One person starts with a short sentence, such as "Once upon a time...", and the next person continues, until the story is finished. Then you can end with snip, snap, snout, and the adventure was over.

TRUE OR FALSE

Each person prepares 3 sentences that describe things about themselves, including 1 true and 2 false sentences. A person starts with the first sentence, and the whole group has to decide what is true or false. The same is repeated for statements 2 and 3. Alternatively, all the statements can be mentioned, and then the group will decide what is true or false. Here you are allowed to make stupid suggestions, as long as one is true!

WHERE DO YOU STAND?

Make a line on the ground where there is the opportunity to stand on both sides. A person asks questions where to stand on one of two sides of the line, depending on what suits best or what they like best. For example: Dog or cat? Here, the person leading the task must clearly show which side is which. Other examples of questions could be TikTok or SnapChat? Summer or Winter? Cozy clothes or nice clothes? Hat or ear warmers? Shoes or barefoot? Salty or sweet? Water park or funfair? The questions must be adapted to the age group.

COMMON AND UNIQUE

Ask questions to find out what they have in common, and differences. Who thinks adults are hopeless at using social media? Who has a pet? Who has siblings? Who eats ribs on Christmas Eve? Pork chops? Red or brown Christmas soda? Summer or winter? Chocolate or licorice?

HELLO GAME – The blue cards can be used to get to know each other better in a smaller group. Suitable for slightly older children/adolescents.

Exercise 1: "Photo-memory"

The purpose of this exercise is to gain an insight into the children's experiences, thoughts and feelings related to their own health service by using visual means (pictures from the Obesity Outpatient Clinic). **Time:** Approx. 30 minutes per subtask, a total of 90 minutes. 5 minutes must be set aside for breaks between tasks.

Preparation:

Three large sheets of paper are hung on the wall, one for each subtask. Prepare three photo series: "Getting to the Obesity Outpatient Clinic", "Weighing and other measurements" and "Conversations with healthcare professionals". Post-IT notes in three different colors, for example: yellow, pink and blue (= other things). Drawing materials and pens in different colors. Tape or school chewing gum to hang the pictures.

Implementation:

The pictures are laid out on the table for each task, and hung up on the respective sheets as they are finished. The children first get 5 minutes separately where they have to write down words on post IT notes for what they think of when they see the different pictures. If they do not want to use personal answers, they can answer what they think other children might think when they come to the Obesity Outpatient Clinic/Hospital for the first time. Those who want to, hang up their own notes in random order and explain these to the others in the group. Those who do not want to talk in front of the group can ask the researcher/youth expert to hang up. 5 min break.

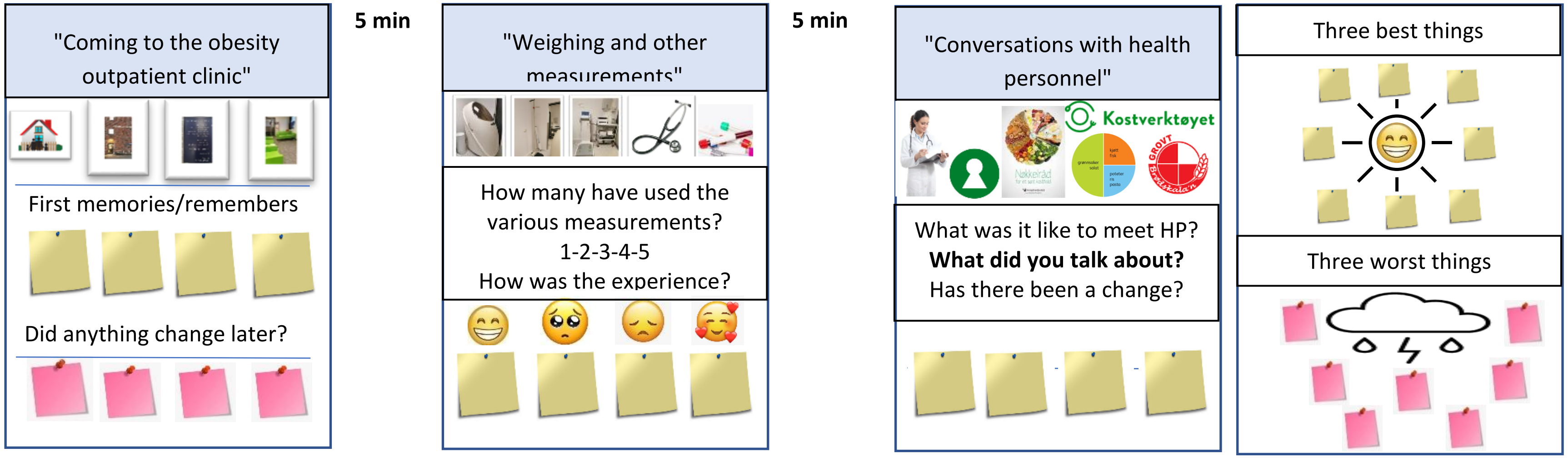
"Coming to the obesity outpatient clinic"
First memories/remembers
Did anything change later?
5 min
"Weighing and other measurements"
How many have used the various measurements?
1-2-3-4-5
How was the experience?
5 min
"Conversations with health personnel"
Kostverktøyet
What was it like to meet HP?
What did you talk about?
Has there been a change?
Three best things
Three worst things

First photo series: "Coming to the obesity outpatient clinic"

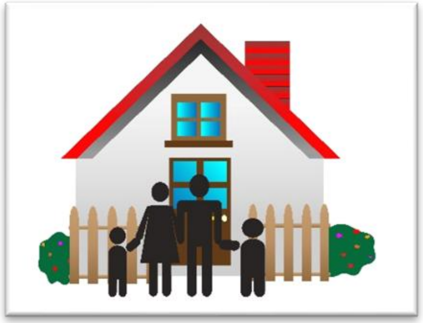

"At home"

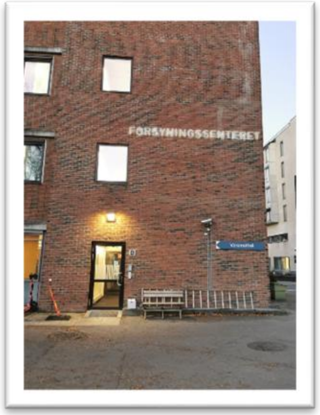

«The hospital»

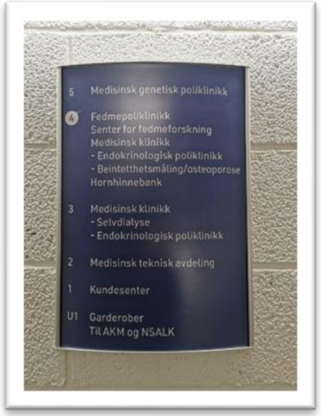

«Sign on the stairs»

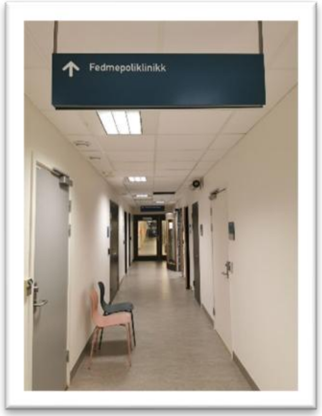

«Obesity Outpatient Clinic»

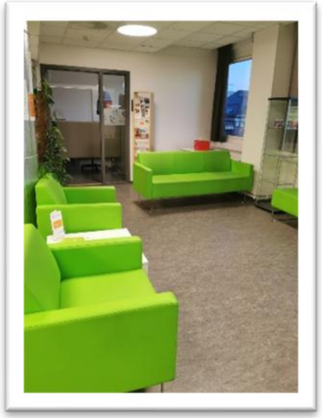

«The waiting room»

FIRST MEETING (25 min)

1. Introduce the exercise (5 min). Now we will do an exercise where you will try to remember what you have been involved in here at the Obesity Outpatient Clinic, and describe what you remember best. Hang up the large sheet of paper and lay it out over the pictures. We have printed some pictures, which we hope will make you remember better. We will ask about things that happened at home before you came here for the first time, about the journey to the hospital, and up until you came here, walked up the stairs, and sat in the waiting room (point to the pictures). We will go through one picture at a time, but first we will show an example, so it will be easy to understand the task.

The group leader refers to an example, by telling a situation where they have sat in a waiting room or waited for a treatment. For example, at the dentist, doctor or others. Write down examples of what you remember best on a note, about both feelings and thoughts, and show what a note might look like. Feel free to stick on an emoji. Say there are no wrong or correct answers, and the kids can either draw, write, or use emoji. Now we will start on the task that applies to you. Everyone is given a stack of yellow notes.

Remembers/remembers best: Yellow notes.

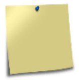

2. Brainstorming 1 (5 Min.) Now we researchers will learn more about what it has been like for you to come to the Obesity Outpatient Clinic for the **first time**? If you don't remember exactly the first time, you can write down what you **remember best**. You can use the pictures to help, but feel free to write down the first thing that comes to mind.

Everyone gets about five minutes each: Write down words on **yellow notes** – everything you thought, and felt, **the first time** you came here, or what you **remember best**. Feel free to write notes for each picture. There may be other things that are important to you, that we have not made pictures of. Write as many notes as you want.

Support questions:

Picture 1: "At home"	Pictures 2-4: "The road there"	Image 5: "The waiting room"
• Did you know why you were going here and what was going to happen? *<br>• Had you talked about it at home before you came here for the first time? *<br>• Do you remember what you had talked about at home before you came there for the first time? *<br>• What do you think the others had thought the first time they came here?<br>• Do you remember if you received a letter? Did you read it? Or was it mostly for the parents?<br>• Did you want to go, or not? Would you like to say anything more about that? *<br>• Did you/the family make any special preparations before the first meeting?	• How did you get to the hospital?<br>• Who did you get along with?<br>• Was it easy to find your way around?<br>• What did you think on the way to the waiting room?<br>• Did you know who you were going to meet? Who, if any, was it?<br>• What do you think of the name of the "Obesity Outpatient Clinic"?	• What do you think it looked like in the waiting room? Was it as you had thought?<br>• Was there anyone else sitting there?<br>• Did you have to wait a long time?<br>• What did you do while you were sitting in the waiting room?<br>• Do you remember what you thought when you sat in the waiting room for the first time?<br>• What is it that you remember best from the waiting room?

3. Hanging up yellow notes. The children (or youth experts) hang the notes on the large sheet of paper and explain. Feel free to hang the notes up near the pictures that fit best. The children can be allowed to ask questions to those who hang up the notes first. The youth experts can also ask questions. The researchers ask any questions at the end. **P.S.** It is allowed to write notes along the way as well, if they come up with something more. When everyone has hung up their notes, we take a new round on each picture, and hear if there is anyone who has come up with something more along the way.

4. Brainstorming 2. Think back on whether there was anything that changed about coming here to the Obesity Outpatient Clinic, after you had been here for the first time. The group leader uses their example further and gives examples of thoughts and feelings related to the next/last time they were going to class.

Things that have changed: Pink patches

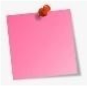

Five minutes: Individual brainstorming. Write down words on **pink notes** – about everything that was different about coming here **after the first time** or after you had been here several times. Write down everything you thought, felt the next time/ the last time you came here. Maybe the pictures can help here too?

5. Hanging up pink notes. The children (or youth experts) hang up notes and explain. Ask the children if they have any questions for those who hang up the notes first. The youth experts can also ask questions. The researchers ask any questions at the end.

Support questions:

Picture 1: "At home"	Pictures 2-4: "The road there"	Image 5: "The waiting room"
• Did anything change at home after you had been here for the first time?<br>• Did you talk about other things at home after you had been here for the first time?<br>• Did you talk more or less before you were going back here?<br>• Did you more or less want to go here? Would you like to say something more about that?<br>• Did you now know more about what was going to happen next?	• How did you get to the hospital?<br>• Who did you get along with? Or did you come alone?<br>• What did you think on the way to the waiting room?<br>• Did you know who you were going to meet? Who, if any, was it?	• What was it like to come to the waiting room after the first time? Was it different from the first time?<br>• Do you remember what you thought when you sat in the waiting room after the first time?<br>• What is it that you remember best from the waiting room now?

Is there any other information that deserves a note? Agree with the children. These must be hung up, but with a separate **color (blue).** These are placed at the bottom of the sheet. Note! No notes on top of each other, and do not hang up notes in order of person.

<u>**Second Picture Series: Weighing and Other Measurements**</u>

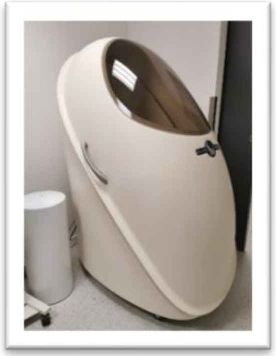
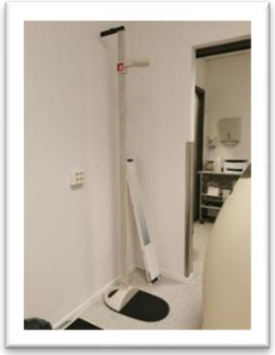
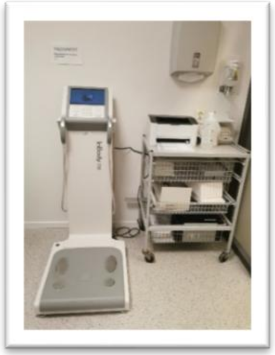
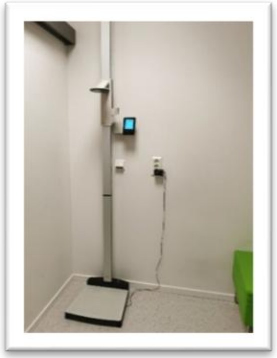
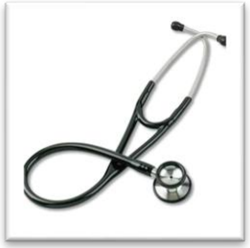
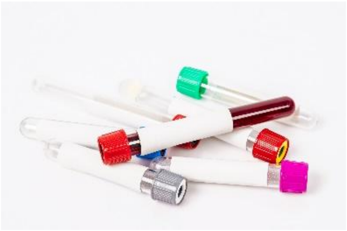

Photos: InBody/ Tanita/BodPod/altimeter/ regular weight/ blood tests/stethoscope/blood pressure monitor

Hang up a new round of posters, take down the previous one. Overlay loose photos.

WEIGHING AND OTHER MEASUREMENTS (25 min)

1. Introduce the task (5-10 min). Explain what the different images/instruments are, and ask which of the children has tried the different measurements. The pictures are placed at the top of the poster, with the correct name of the measuring instrument. Two columns on the large sheet: 1 larger column for the children's notes (yellow notes), and a smaller column for other things (blue notes).

Write down figures for how many people have taken the various measurements. Has anyone tried any of these several times? Other measurements/instruments we have forgotten? The researcher can tell about their own experience with blood tests or measuring/weighing first (only if the group is quiet and there is a need). The participants are then asked to think back to the time they took one or more of these measurements. **What were they thinking, and what was it like for them? (Emoji = emotion)**

2. Five minutes: Individual brainstorming. Write down key words on **yellow notes**.

3. Ask the children to hang the note around the picture in question and explain (i.e. key words if the Bod Pod is hung around this picture) + write which measuring instrument it applies to at the top of the note. Remember to ask one more time if anyone has had new thoughts or notes along the way. Blue notes for other things that come up.

Support questions:

• *Did you know in advance that you were going to take these measurements?*<br>• *Who took the poll(s)? Did you get an explanation as to why?*<br>• *Where were they made? How was it for you? What did you think?*<br>• *Someone caught several times, better or worse next time?*	• *What do you think about these different measurements? Good? Less good?*<br>• *Which polls did you like best/worst?*<br>• *Difference in the polls from the first time to the next/last?*<br>• *Did you want to be measured? Or not?*

Third photo series: Conversations with healthcare professionals

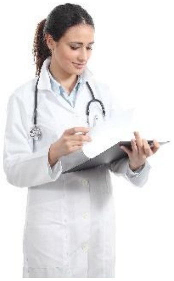
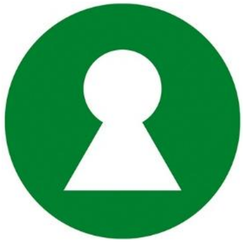
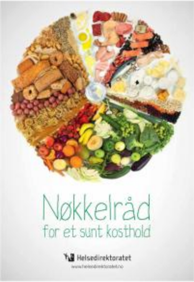

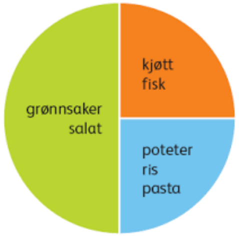

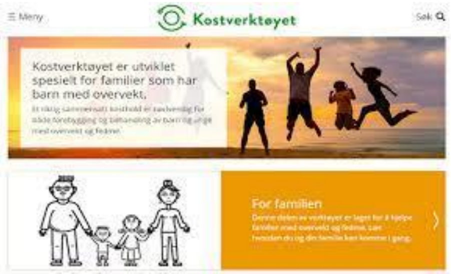

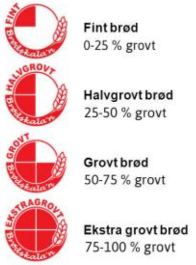

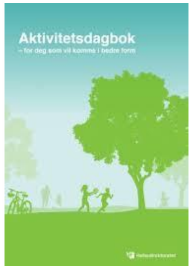

Images: Healthcare personnel / **Food diary** / Diet tool / Image bank / Keyhole label **/ Daily menu** / Tasting chart / Dietary circle ++

1. Introduce the task (2-3 min): Now we will do an exercise that will be a little different. Now you will discuss things in groups, and eventually you will see some pictures of things that may have been used during the conversations you have had with health personnel. Now it is we (researchers or youth experts) who write the notes, but everyone must agree on what should be on the notes and which notes should be hung up. Finally, you will be given the task of sorting the notes according to which ones you think belong together.

Communication: *How were you met?* ***(5-10 min)***

So far, we have talked about things that have happened at home, on the way here and in the waiting room. Now we researchers are curious about what has happened after you have sat in the waiting room, and what it has been like for you to come here and talk to the health personnel who work here? We can start with everyone trying to remember the times they have been here: Do you remember who you met the first time? Was it a man or a woman? Have you met the same person every time? Did you meet physically or digitally? Did they talk to you or your parent(s)? Do you remember what was talked about? Did you understand what was said? Did you ask what you were wondering about? Give an example if no one says anything/when necessary. Explain that those who work here do not want to know who has said what, and that they want to know both what you think is good, but also if there is anything that has been said or done that you do not like, or think can be done differently.

Is there anyone who would like to say something about what it has been like to talk to health personnel? (Tove's suggestion: dice roll 1-6 or emoji for general experience → justify the choice). The children talk, researchers write down notes, and a youth expert or researcher hangs up the note.

Support questions:

• *Who did you meet for the first time? Who have you met the other times? (same person or different)*<br>• *What was it like to meet him/she/these? Was it nice, scary or something else?*<br>• *Did he/she talk to you or your parents?*<br>• *Did you understand what he/she/they were talking about?*	• *Did you ask about things you were wondering about? Why, why not?*<br>• *Did anyone talk about why you were there?* (Has anyone explained to you why your body is heavier than many other children?)*<br>• *How was that explanation? What did you think then?*<br>• *Did you meet physically or digitally?*

Content: *What do you remember from what was talked about?* ***(5-10 min)***

We are now putting **various pictures** on the table of typical things that we know healthcare personnel use in conversations with children and families. *Do you recognize any of these? Which ones have you used?* The children explain what they have used, and discuss this together. The researcher asks relevant follow-up questions on an ongoing basis and notes key words on post-IT notes as the children tell their stories.

Support questions:

• *Are there any of these that you have liked better than others?*<br>• *Anyone you haven't liked?*<br>• *Is it something you or your family have used at home?*<br>• *Are there other important things that we have not taken pictures of?*	• *What other things have you talked about, in addition to what is shown in these pictures?*<br>• ***Is there any advice about body and health that you think can make things worse, and is there advice that can make things better? Why do you think that?***

Change**: Has anything changed since you've been here?* ***(5-10 min)

1. Place a large sheet of sun for positive change + rain cloud for negative change across the table. Now we want you to use yellow patches for what has improved (been good) by being here at the Obesity Outpatient Clinic, and pink patches for what has gotten worse (been less good). Then we ask the children to stick up the yellow notes around the sun and explain + the pink notes under the rain cloud with explanation.

Support questions:

• *Is there anything that has improved about being here?*<br>• *What was the best thing about being here?*<br>• *Have there been any changes to you?*	• *Was there anything that was less good about being here?*<br>• *What did you like least about being here?*<br>• *Have there been any changes at home (with parents or siblings)?*

Alternatively: One person asks questions and writes a note (group leader) and one person ('research assistant') hangs up the note, while the children discuss. Older children can group the post-IT notes by theme. Decide what the topic should be called. The younger children need more help, and should not group all by themselves.

Follow-up work: The group leader summarizes a presentation of the results from this exercise, which will be presented to the others in the group at the end.

Alternative exercise for younger children (9-12 years): Role play

Preparations/equipment: White coat etc. (soft toy or other items), dress up clothes for parents (suit jacket/shawl/handbag)

In this assignment, we will have a role-play. We can imagine that it is a family that comes to the obesity outpatient clinic for the first time for a conversation with their parents. Is there anyone who wants to join? We will do it together, but in a slightly different way. We take turns, so that children become adults and adults become children. If more children want to join, they can be siblings or pets.

Show an example first when needed, for example from a dentist visit. We adults show, using humor.

Now we need a child who can play the role of health personnel and someone who will play the parent. The researcher plays the child. We present various things that can typically be used in such a conversation, and the children can choose completely free to use what you want, or possibly pretend that you use something completely different, or not use anything at all.

Intro:

Imagine that you are a health professional, imagine that you are going to have a first conversation with a child. First you have to weigh and measure the height, and then talk. Here you are free to talk about what you want, but feel free to bring something you remember from when you were at the conversation yourself, or it may be something you may wish it was talked about. The rest is the audience.

Let several children try different roles, if they wish. They can also be children if they want. Anyone who wants a role can join. Some may also be extras. And the researchers can also be told not to participate.

Finally, when everyone is done:

- What do you think of this assignment? What was it like to have role-playing?
- What was it like to be a healthcare professional? Or parents?
- Be open to what is happening, and bring up things that may come up that may be interesting to discuss together.

Exercise 2: "Body image"

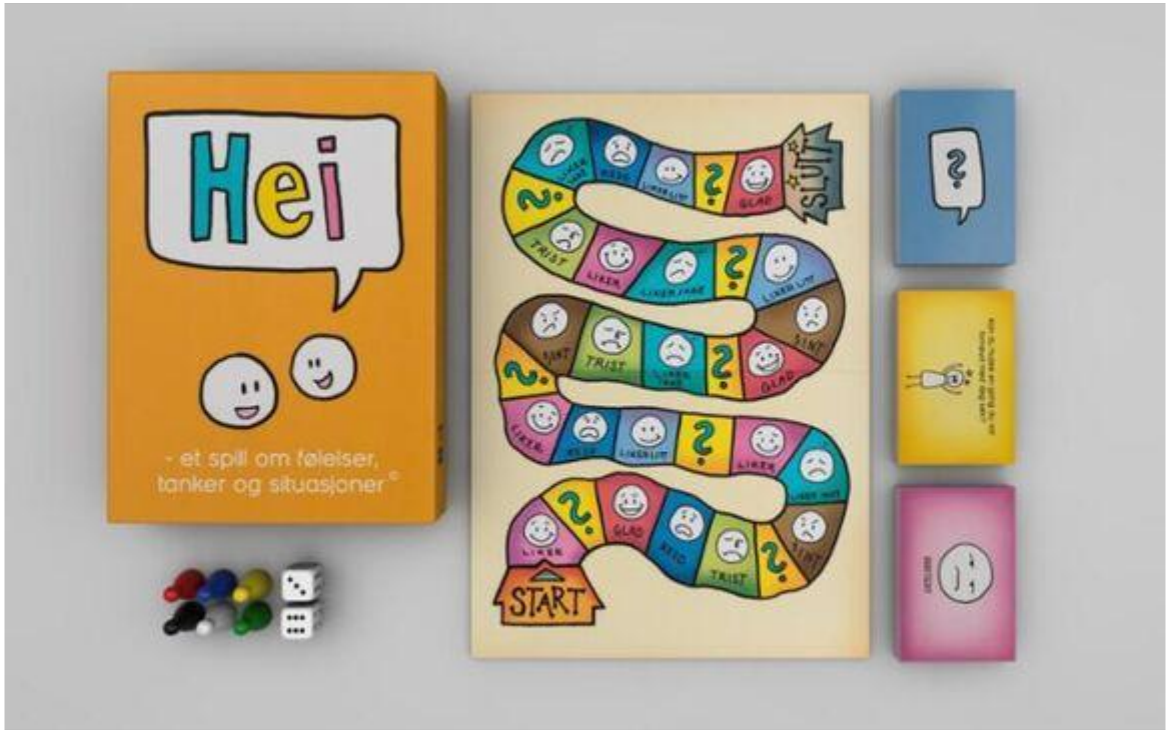

Introduction (5 minutes)

Now you will try the Hello game, a game about feelings, thoughts and situations. We want to test whether it is possible to play this by using examples from what you have been involved in here at the Obesity Outpatient Clinic. Has anyone seen or tried this game before? Anyone who wants to try?

Game rules: Everyone chooses their own color and turns on START. Use either dice 1-6 (for younger children) or dice 1-3 (for older children). If you land on a face, you should describe something that you associate with this feeling. For example, if you land on the smiley face (= like), then you should describe something you like. If you land on a question, you must draw a question card and try to answer various questions that are about thoughts, feelings and the body.

Background (for researcher): All the questions will be about different aspects of body image, and the questions will be explored in different ways (feelings about the body/thoughts about the body/perspective/behaviour as a result of feelings/thoughts related to the body).

Example: Roll the dice (1-3) and move either:

- 1 (Smiley face = Like) → Say something you **like** by participating in the offer of the Obesity Outpatient Clinic.
- 2 (Question card) → "A lot of people don't like gym, why do you think that's the case?"
- 3 (Smiles = Happy) → Say something that makes you **happy** by being at the Obesity Outpatient Clinic.

It is allowed to answer do not know, or ask if there is anyone else in the group who can help. Remember that the best answer is often the first one that comes to your head, and here there are no answers that are wrong.

Start the Hello Game (45 minutes)

The poster with the body silhouette is hung up. For every time a player solves a task, we hang the answer on a post-IT note **on** the body silhouette. In the case of question cards, we hang up the question card with the answer **around** the body silhouette. It is allowed to use emojis to describe emotions. Put on a stopwatch, and let the last person finish.

Joint review (10 minutes)

Finally, the group can discuss together the cards and experiences that we have hung up. Take one question card or experience at a time (post IT note), and ask the group if they have any more suggestions? Hang several post-IT notes around. If there are still some question cards left, and there is extra time, these can now be addressed together.

We're going to do a little twist on this game, but first I'm going to tell you a story about a boy named Petter (and a girl named Emma).

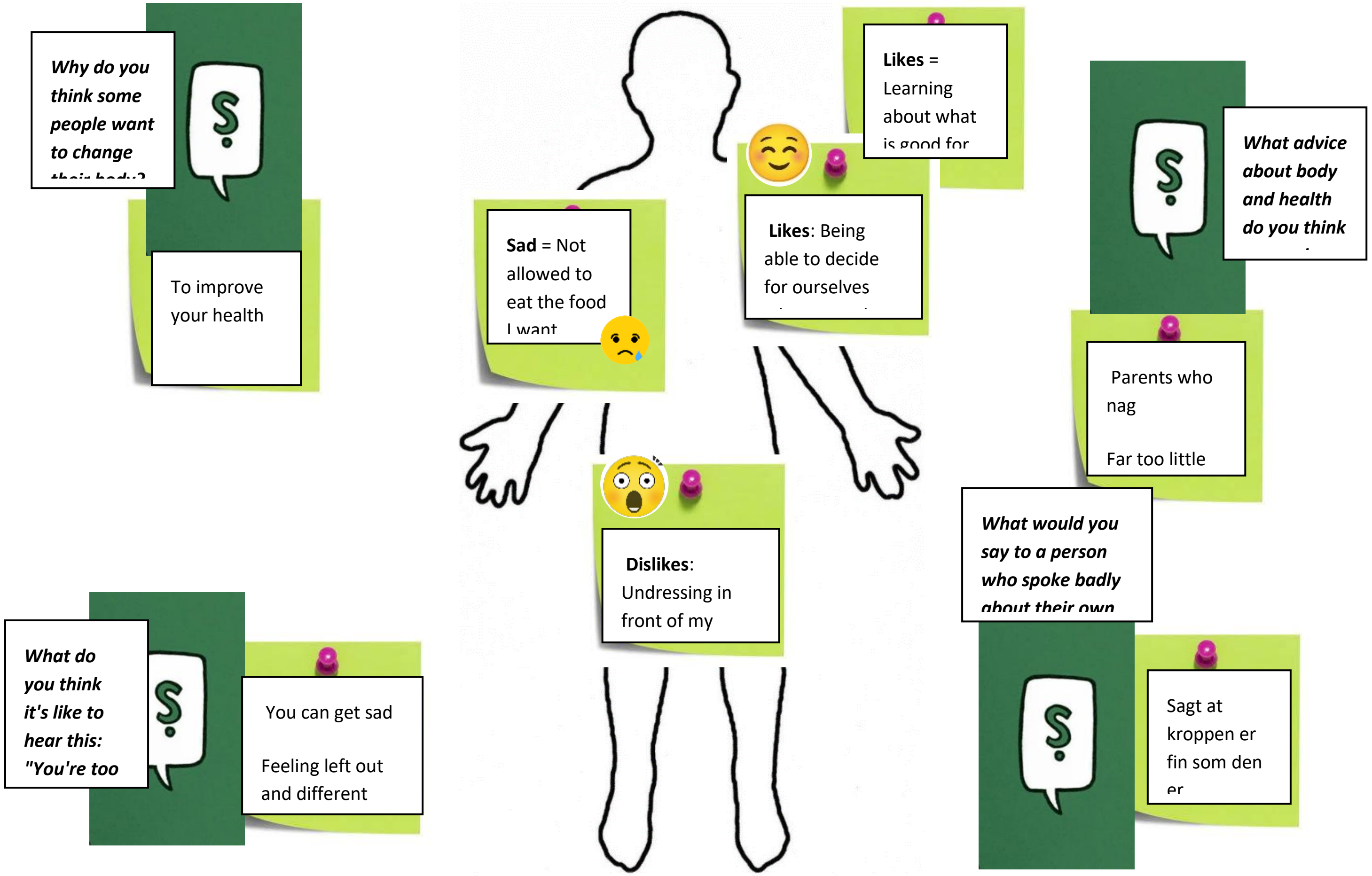
Why do you think some people want to change
To improve your health
Sad = Not allowed to eat the food I want
Likes = Learning about what is good for
Likes: Being able to decide for ourselves
What advice about body and health do you think
Parents who nag
Far too little
Dislikes: Undressing in front of my
What would you say to a person who spoke badly about their own
What do you think it's like to hear this: "You're too
You can get sad
Feeling left out and different
Sagt at kroppen er fin som den er

Current questions about body image:

- What do you think it's like to hear this: "You're too big!" (shame)
- What do you think it is like to hear this: "You are too small!" (shame)
- What do you think it's like to hear this from others? "It's just a matter of pulling yourself together" (shame)
- Many people don't like gym or exercise. Why do you think this is so? (function)
- What would you say to a person who spoke badly about their own body? (brown card)
- What would you say to a person who spoke badly about other people's bodies? (made themselves)
- Why do you think some people want to change their body? (pressure of expectations)
- What do you think most boys/girls want to look like? (pressure of expectations)
- How important is it to look like each other in order to fit in? (pressure of expectations)
- What influences our thoughts about what the body should look like? (pressure of expectations)
- How important is it to have a lot of muscle? (pressure of expectations)
- What advice about body and health do you think can make things worse? (pressure of expectations)
- What advice about body and health do you think can make things better? (made themselves)
- What is important for a person to feel good about their body (regardless of size)? (made themselves)

Exercise 3: Ranking exercise

The purpose of this exercise is to compile the most important results from the two previous exercises and discuss this within the groups. Furthermore, everyone must present the results to each other.

Time: Approx. 60 minutes.

Preparation:

Hang up blank forms for the various tasks, with recognizable pictures that the children will recognise. New post IT notes and pens.

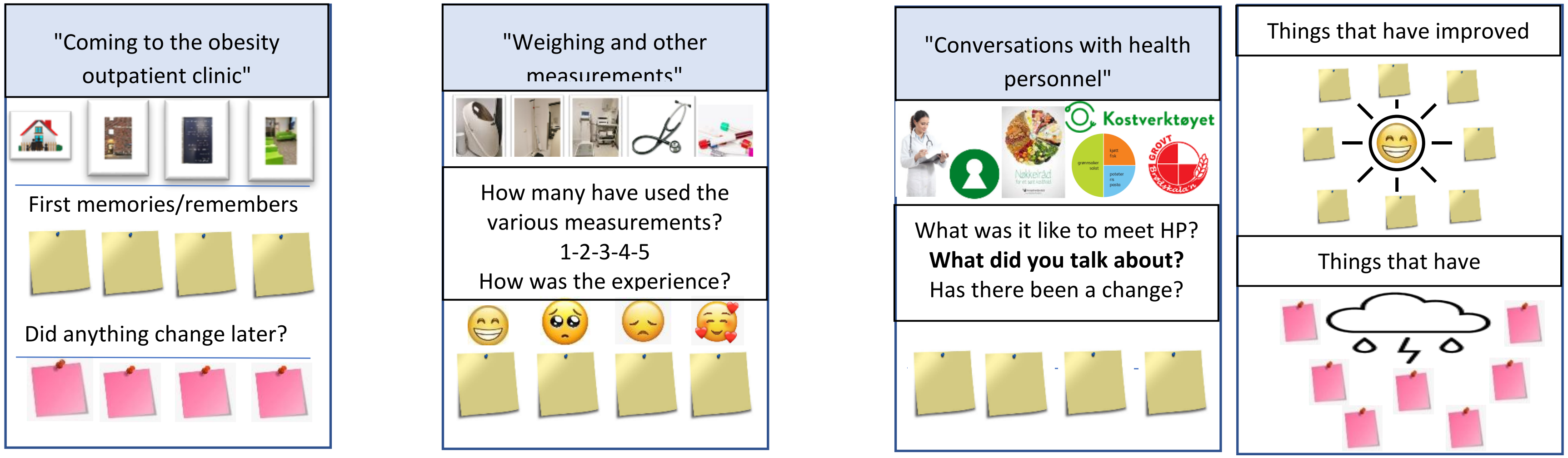

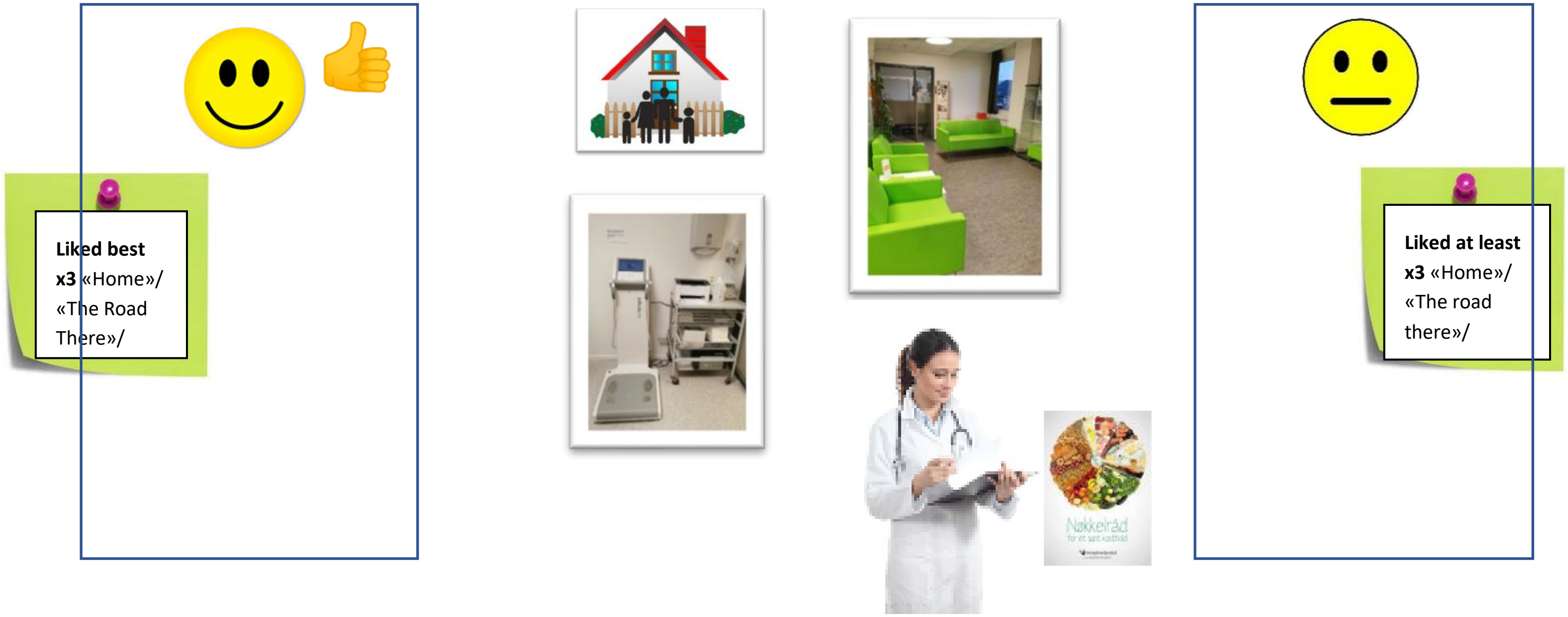

1 All the children write down the **three** most important things that are good at the Obesity Outpatient Clinic (yellow/green notes) and **three** suggestions for improvements (pink), linked to the three different picture series:

- Getting to the Obesity Outpatient Clinic 3+3 (home, the road there and the waiting room) –

Help question: What are smart things parents can say or do to prepare you for what is going to happen? How

- Weighing & Measuring 3+3
- Health professionals
    - Communication 3+3
    - Content 3+3
    - Change 3+3
- Thumbs up and thumbs down from the game

Can the group leader or youth write down?

Age adjustments for the youngest children, on this last task.

Exercise 2: Body image

- 3 things about your body and health that can make things better
- 3 things about your body and health that can make things worse

Exercise 4: Joint presentation

The purpose of this exercise is to achieve a joint summary from all the groups, as well as a review and validation of the findings together with the informants.

Time: Approx. 60 minutes.

Preparation:

The responsible researcher from each group creates a joint summary of the results for their own group, as well as a word cloud. The template for the PowerPoint presentation is prepared in advance. Content is recorded during the breaks after each subtask. Arrange a printout of the PP presentation for the children, so they can watch at the same time.

Implementation:

1. Each group presents its results to each other in chronological order according to the tasks we have gone through (Waiting room / Weighing and measuring / Healthcare personnel). All children are encouraged to come forward. Those who want to can present. If no one wants to, the researcher responsible for that group can present. Reuse the same pictures so that the children recognize the different tasks. Set aside up to 10 minutes per group.

2. After all the groups have presented their results, we look at the similarities and differences between the findings in the different groups:

- Are there any common features?
- Differences between the groups? Possibly between different age groups?
- What do the children think of these results? Any thoughts? Can this be presented to politicians, hospital management and others?
